# Kinetochore-associated protein 1 promotes the invasion and tumorigenicity of cervical cancer cells via matrix metalloproteinase-2 and matrix metalloproteinase-9

**DOI:** 10.1080/21655979.2022.2061144

**Published:** 2022-04-07

**Authors:** Caimei Wang, Yiyuan Wang, Congrong Liu, Xiaoyu Meng, Zhongxia Hang

**Affiliations:** a Obstetrics and Gynecology, Yulin Second Hospital, Yulin, Shaanxi Province, China; b Department of Stomatology, The Second Affiliated Hospital of Shaanxi University of Traditional Chinese Medicine, Xianyang, Shaanxi Province, China; c School of Medicine, Xi’an Jiaotong University, Xi’an, Shaanxi Province, China

**Keywords:** Cervical cancer, KNTC1, invasion, tumorigenicity, MMP-2, MMP-9

## Abstract

Cervical cancer, a common cancer in women, has become a serious social burden. Kinetochore-associated protein 1 (KNTC1) that regulates the cell cycle by regulating mitosis is related to the malignant behavior of different types of tumors. However, its role in the development of cervical cancer remains unclear. In this study, we initially explored the role of KNTC1 in cervical cancer. KNTC1 expression and relevant information were downloaded from The Cancer Genome Atlas (TCGA) and dataset GSE63514 in the Gene Expression Omnibus (GEO) database for bioinformatics analyses. Cell proliferation was detected by cell counting kit-8 (CCK8) and colony formation assays. Wound healing and Transwell assays were used to evaluate cell migration and invasion abilities. Protein expression levels of matrix metallopeptidase 2 (MMP2) and matrix metallopeptidase 9 (MMP9) were measured by western blotting. Nude mouse models of subcutaneous xenograft tumor were constructed to analyze tumor growth in vivo. CCK8 and colony formation assay results demonstrated that the proliferation rate of SiHa and C-33A cells decreased when KNTC1 was silenced. Western blot and Transwell assays indicated that KNTC1 knockdown weakened the invasion and migration abilities of SiHa and C-33A cells and decreased the expression of MMP-2 and MMP-9. In-vivo experiments suggested that the inhibition of KNTC1 reduced tumor growth. Taken together, our study showed that KNTC1 plays an important role in cervical cancer. Further, we verified the promotional effect of KNTC1 on cervical cancer through in-vivo and in-vitro experiments and speculated that KNTC1 might mediate tumor invasion via MMP9 and MMP2.

## Graphical abstract

**Figure:**
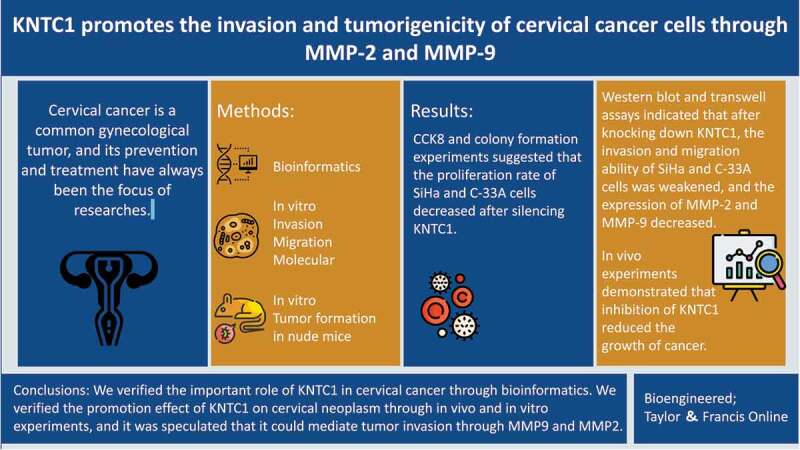

## Introduction

Cervical cancer is one of the most frequently observed tumors in women [1] and is a global public health concern that needs to be addressed urgently. In recent years, the incidence of cervical cancer has declined in developed countries due to human papillomavirus (HPV) vaccination; in developing countries, however, it is still increasing owing to the lack of screening or prevention. In China, due to the increase in both incidence and mortality, cervical cancer has become a common disease, especially in young women [2]. An estimated 570,000 women were newly diagnosed with cervical cancer in 2018, and 313,000 died of cervical cancer [3]. Infection with high-risk human papillomavirus is one of the key risk factors for cervical cancer; 90% of HPV infections regress within two years, and very few develop into cervical squamous intraepithelial lesions. HPV vaccination can reduce infection rates and prevent cervical cancer to some extent; however, it is not widely available, especially in developing countries. Therefore, the number of patients with cervical cancer continues to increase [4]. Cervical cancer poses a heavy economic burden to both family and society. Therefore, it is particularly important to elucidate the molecular mechanisms underlying cervical cancer and identify the possible therapeutic targets.

Kinetochore-associated protein 1 (KNTC1), sometimes referred to as the rough deal (ROD), is an important component of the mitotic checkpoint. Small mistakes in mitosis can lead to disastrous consequences in cells. During mitosis, kinetochores must bind to and maintain dynamic connections with the microtubules from the spindle to guide chromosome separation. In addition to moving chromosomes, kinetochores must monitor and correct the process to ensure an accurate separation. Kinetochores are composed of more than 100 proteins and their ordered assembly is very important [5]. Kinetochore proteins usually exist in the form of a complex. Aberrant expression or assembly of a single kinetochore protein can affect the complex formation and centromere localization, increase chromosome instability, lead to inaccurate chromosomal separation and aneuploid cells, and ultimately promote tumorigenesis [6]. Studies have shown a variety of kinetochore proteins to be associated with tumors. Kinetochore-associated protein 2 (KNTC2), also known to be highly expressed in cancer 1 (HEC1), is elevated in gastric cancer, colorectal cancer, prostate cancer, and many other tumors; tumor growth is inhibited when KNTC2 is downregulated [7–9]. Abnormal expression of the spindle assembly checkpoint proteins mitotic arrest deficient 1 and 2 (Mad1 and Mad2) is also involved in tumor formation. Downregulation of Mad1 or upregulation of Mad2 increases chromosomal instability and promotes aneuploidy and tumorigenesis [10,11]. Kinetochore scaffold 1 (KNL1) has been confirmed to be associated with the malignant behavior of colorectal cancer, lung adenocarcinoma, gastric cancer, and other tumor cells [12–14]. Spindle and kinetochore-associated (SKA) family members play a vital role in breast cancer. Spindle and kinetochore-associated complex subunit 1 (SKA1) can promote oxaliplatin resistance in breast cancer cells by activating the Notch and Wnt pathways [15]; inhibition of spindle and kinetochore-associated complex subunit 2/3 (SKA2/3) attenuates proliferation and migration of tumor cells [16].

As a core kinetochore protein, KNTC1 prevents cells from exiting mitosis prematurely, maintains accurate replication and separation of genomic DNA, and is essential for guaranteeing orderly implementation of mitosis. Currently, there are very few studies on KNTC1. KNTC1 has been reported as a key gene of rheumatoid arthritis [17] and polycystic ovary syndrome [18], and may be involved in the occurrence and development of different tumors. Bioinformatics analyses have shown remarkable expression of KNTC1 in cervical cancer tissues, which has been confirmed by immunohistochemistry; moreover, KNTC1 has been reported to be elevated in the blood of patients with cervical cancer [19–21]. However, its role in the development and progression of cervical cancer still remains unclear.

Matrix metalloproteinases (MMPs) form a family of zinc-dependent endopeptidases that represent the most important protease family related to tumorigenesis. Their main biological function is to degrade the extracellular matrix and participate in the regulation of various cell behaviors, such as proliferation, migration, apoptosis, differentiation, and angiogenesis [22]. MMPs are overexpressed in many cancers, and their relationship with malignancy has been reported in previous literatures. MMP-2 and MMP-9 are associated with the clinical stage and lymphatic metastasis of laryngeal cancer. Their high expression is a predictor of poor prognosis of laryngeal cancer, and is associated with invasion and metastasis [23]. For primary cutaneous malignant melanoma, high levels of MMP-2 are independent biomarkers of disease recurrence and overall survival [24]. MMPs also play an important role in cervical cancer, and are associated with the degree of malignancy. MMP2 is associated with a higher risk of death from cervical cancer [25]. The relative activity of MMP2 and MMP9 increases gradually from normal cervical tissue to squamous intraepithelial lesions and then to squamous cell carcinoma, and is significantly correlated with disease stage [26].

To initially explore the pathogenic role of KNTC1. In this study, we hypothesized that KNTC1 promotes the development of cervical cancer, and explored the underlying mechanism by cell culture and animal experiments. The effectiveness of KNTC1 as a tumor therapeutic target will be further explored in subsequent studies.

## Materials and methods

### Bioinformatics analysis

Statistical analysis and bioinformatics data visualization were conducted through R. Cervical cancer GSE63514 series from the Gene Expression Omnibus (GEO, https://www.ncbi.nlm.nih.gov/geo/) database was obtained using the GEOquery package to further organize and standardize the data [27]. Subsequently, the limma package was used for differential gene analysis [28], and the clusterProfiler package was used for gene ontology (GO)/Kyoto Encyclopedia of Genes and Genomes (KEGG) analysis [29]. The ComplexHeatmap package presented the expression of top 20 genes with high and low expression in the expression profile. The level 3 HTSeq-FPKM format RNAseq data and clinical data from the cervical squamous cell carcinoma and adenocarcinoma (CESC) project of The Cancer Genome Atlas (TCGA) (https://portal.gdc.cancer.gov/) were used to analyze the expression of KNTC1 [30]. The pROC package was used to draw the ROC curve and determine the discrimination efficiency of KNTC1. The survminer and survival packages were used to draw Kaplan-Meier survival curves.

### Cell culture and transfection

Cervical cancer cell lines SiHa and C-33A, and the human normal cervical epithelial cell line HcerEpic, were purchased from Procell (Wuhan, China). SiHa and C-33A were cultured in minimum essential medium containing 10% fetal bovine serum, and HcerEpic cells were cultured in complete medium for human cervical epithelial cells provided by Procell. All the cells were maintained in a humidified incubator containing 5% CO_2_ at 37°C.

C-33A and SiHa cells were seeded in 6-well plates, and when the cell density reached 40%, 60 μl lentivirus with a titer of 10^8^ TU/ml was added. The sequences of short hairpin RNA (shRNA) targeting KNTC1 (shKNTC1) and negative control shRNA (shCtrl) were as follows: shKNTC1, 5ʹ-TGAGTTTATGGGATATTTA-3ʹ; shCtrl, 5ʹ-TTCTCCGAACGTGTCACGT-3ʹ [31]. The KNTC1 lentivirus and negative control lentivirus were synthesized by Genechem (Shanghai, China). After 2 weeks of puromycin selection, qRT-PCR and western blotting were used to verify knockdown efficiency.

### Cell counting kit – 8 (CCK8) assay

CCK8 assay was used to determine the cell proliferation rate. Cervical cancer cells transfected with lentivirus were seeded in 96-well plates at 3000 cells per well, placed in a 37°C incubator containing 5% CO_2_, and tested at 0 h, 24 h, 48 h, and 72 h. Cell proliferation was measured using absorbance at 450 nm, after they were incubated with a 10% CCK8 solution at 37°C for 2 h.

### Colony formation analysis

C-33A and SiHa cells transfected with lentivirus were seeded in 6-well plates at 1,000 cells per well and cultured in an incubator containing 5% CO_2_ at 37°C. Two weeks later, cells were fixed with 4% paraformaldehyde, stained with crystal violet, and compared.

### Transwell analysis

Cervical cancer cells transfected with lentivirus were resuspended in serum-free medium, and 30,000 cells per well were cultured in the upper chamber of Transwell in a 24-well plate, with complete medium added in the lower chamber. After 24 h, cells were fixed with anhydrous methanol for 15 min and stained with 0.1% crystal violet for 15 min. Upper layer of the cells was wiped off. The chambers were washed with phosphate-buffered saline (PBS), and then observed and counted under an inverted microscope. Transwell migration and invasion experiments were performed in a Transwell chamber with a pore size of 8 μm (Millipore, USA). The invasion experiment was performed similarly, but the upper chamber was pre-coated with Matrigel (BD Biosciences, USA).

### Wound healing assay

C-33A and SiHa cells transfected with lentivirus were seeded in 6-well plates. When cells fused completely, a pipette tip wiped the cell layer to form a straight-line scratch. The C-33A and SiHa cells were washed with PBS and cultured in a serum-free medium. An inverted light microscope was used to observe the distance between scratches in different groups at 0 h and 24 h.

### Western blot analysis

After washing the cells twice with pre-cooled PBS, radioimmunoprecipitation assay (RIPA) lysis buffer containing 1 mM phenylmethylsulfonyl fluoride (PMSF) was added to lyse the cells on ice. A bicinchoninic acid (BCA) assay kit was used to measure protein concentration. Protein samples were separated using 10% sodium dodecyl sulfate-polyacrylamide gel electrophoresis and transferred to polyvinylidene fluoride membrane (Millipore, USA). After blocking with 5% skim milk at about 26°C room temperature for 1 h, the membrane was incubated overnight at 4°C with anti-KNTC1 (1:200, sc-81,853, Santa Cruz, USA), anti-MMP2 (1:1000, 10,373-2-AP, Proteintech, China), anti-MMP9 (1:1000, 10,375-2-AP, Proteintech, China), and anti-GAPDH (1:10,000, 10,494-1-AP Proteintech, China). The membrane strips were incubated with horseradish peroxidase (HRP)-labeled secondary antibodies (goat anti-rabbit, 1:10,000, AB0101, Abcam; goat anti-mouse, 1:10,000, AB0102, Abways) for 1 h at room temperature and then visualized using an enhanced chemiluminescence (ECL) kit.

### RNA extraction and real-time PCR analysis

According to the manufacturer’s instructions, we used TRIzol (Invitrogen) to extract total cell RNA, PrimeScript™ RT Master Mix (RR036A, TaKaRa) kit for reverse transcription, and SYBR® Premix Ex Taq™ I (RR820A, TaKaRa) reagent to perform qPCR on the BioRad CFX96 system. The primers were synthesized by Sangon Biotech (Shanghai, China) and their sequences were as follows: KNTC1, forward 5-TCCCATCGCAGGACGAAAAA-3′ and reverse 5′-ATGTGCTGGCTTTCCGATCA −3′; GAPDH, forward 5′- CCCCACCACACTGAATCTCC −3′ and reverse 5′- GTACATGACAAGGTGCGGCT-3′.

### Animals and treatment

Six female BALB/c-nu/nu mice, 4–5 weeks old, were purchased from the Shanghai Laboratory Animal Company, and raised under specific pathogen-free conditions at the Animal Experiment Center of Xi’an Jiaotong University. The mice were randomly divided into two groups and shKNTC1 C-33A or shCtrl C-33A cells were injected into the left and right armpits of each group. SiHa cells transfected with the lentivirus were injected into the remaining group of nude mice. Length and width of the tumors were measured every 10 days, and volume was calculated according to the following formula: volume = length × width × width/2. Thirty days later, the nude mice were euthanized by cervical dislocation after being anesthetized and the tumors were excised and weighed. Animal experiments were performed according to the experimental guidelines and were approved by the concerned ethics committee of YuLin second hospital.

### Statistical analysis

All experiments were performed in triplicate. Statistical analysis was performed using GraphPad Prism version 8.0 (USA), and data are expressed as the mean ± standard deviation. Student’s t-test and ANOVA were used to compare the differences between the experimental group and the control group, and p < 0.05 was considered statistically significant.

## Results

In this study, we hypothesized and verified that KNTC1 participates as an oncogene in the development of cervical cancer. Based on cell and animal experiments, we concluded that KNTC1 can enhance the invasion, migration, and tumorigenicity of tumor cells via MMP2 and MMP9, providing a basis for the subsequent study of KNTC1 as a target for cervical cancer treatment.

### Bioinformatics results

**Figure 1.**
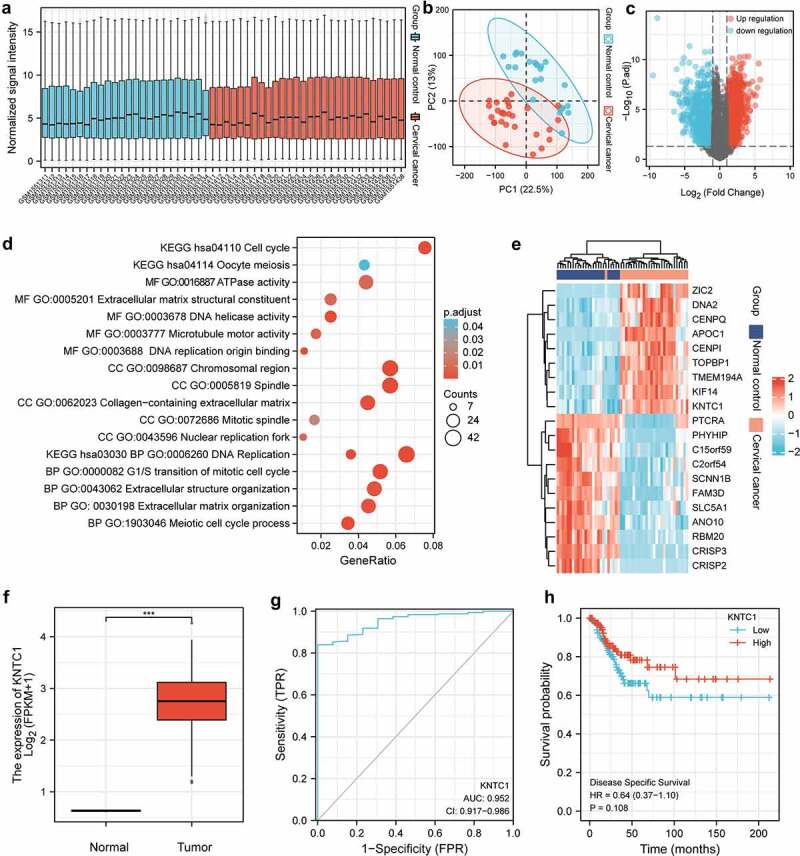
Bioinformatics analysis. (a) Box plot shows the normalized processing results for each sample included in the analysis. (b) Principal component analysis of normal group and tumor group. (c) Gene probe volcano map of normal group and tumor group. (d) GO/KEGG enrichment map of differential genes. (e) Heat map of top 20 differential genes with high expression and low expression in the expression profile. (f) Box plot of KNTC1 expression in normal and tumor group of TCGA database. (g) ROC analysis of the expression level of KNTC1 in distinguishing patients with cervical cancer. (h) KM survival curve for analysis of the prognostic value of KNTC1 in patients with cervical cancer.

In the box plot, median of each sample was on a horizontal line, indicating that the degree of normalization across samples was appreciable (Figure 1a). Samples of the normal and cancer groups were separated, and the ratios of PC1 and PC2 were found to be high, indicating that the differences between groups were obvious, and that subsequent difference analysis might yield more meaningful results (Figure 1b). In the volcano map, 2856 genes met the threshold of |log2(FC)|>1 and p.adj < 0.05. Under this threshold, there were 1804 genes that were highly expressed in the tumor group, and there were 1052 genes that were highly expressed in the normal group (Figure 1c). In the GO/KEGG enrichment map, the entries mainly focused on mitosis, cell cycle, DNA replication and separation, and extracellular matrix reconstruction (Figure 1d). A heatmap was used to visualize the expression of top 20 differential genes with high and low expression in the expression profile. The genes with low expression in the tumor group were CRISP3, FAM3D, SCNN1B, CRISP2, RBM20, PHYHIP, C2orf54, SLC5A1, PTCRA, C15orf59, and ANO10. The most highly expressed genes in the tumor group were TOPBP1, CENPI, KNTC1, CENPQ, TMEM194A, KIF14, DNA2, APOC1, and ZIC2 (Figure 1e). Wilcoxon rank sum test results showed that the expression level of KNTC1 in tumor group was higher than normal, and the median difference between the two groups was 2.694 (2.081–3.311), which was statistically significant (P = 0.003) (figure 1f). In predicting the outcome of normal and tumor tissues, the predictive ability of KNTC1 had higher accuracy (AUC = 0.952, CI = 0.917–0.986) (Figure 1g). The Cox regression results indicated no statistically significant difference in survival time distribution between the groups (Figure 1h).

### KNTC1 expression was upregulated in cervical cancer cell lines

**Figure 2.**
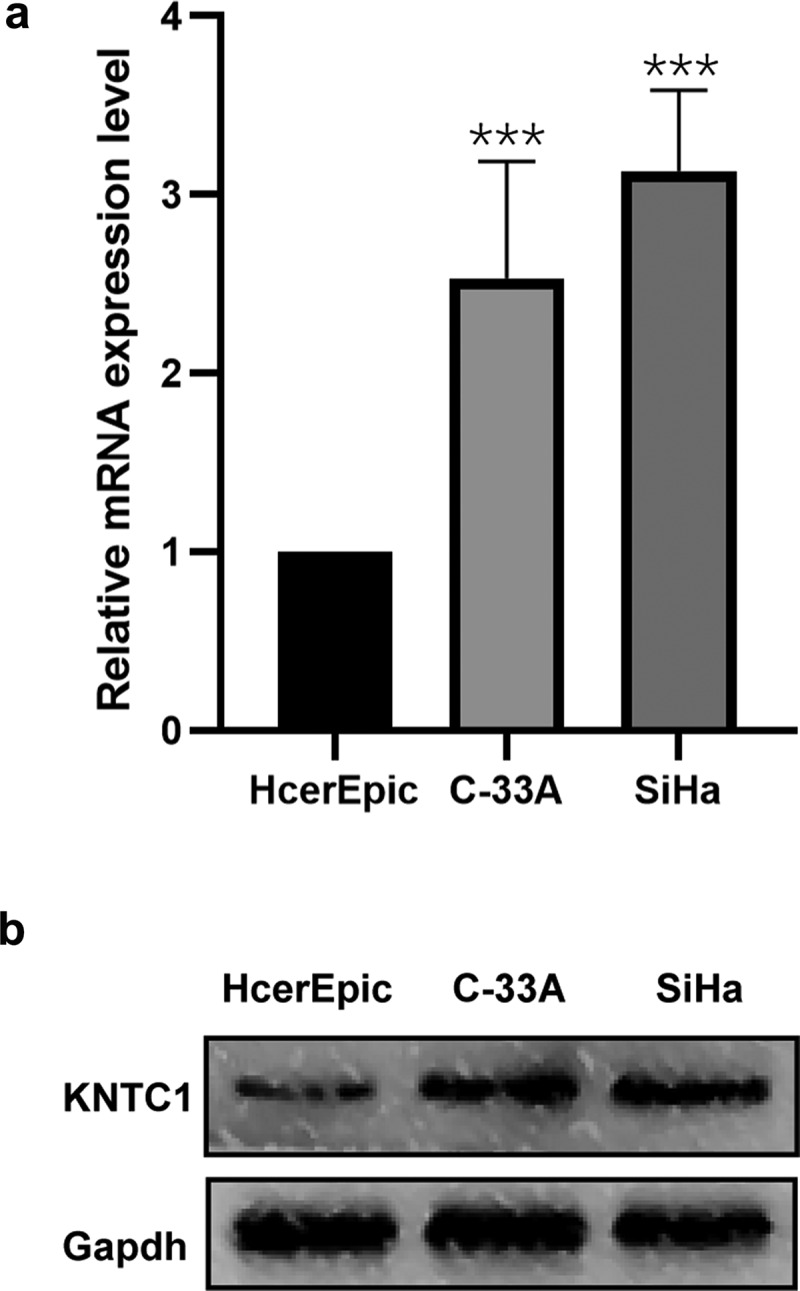
Expression of KNTC1 in cervical cancer cell lines. (a) qRT-PCR detected the mRNA expression level of KNTC1 in human normal cervical epithelial cells (HcerEpic) and cervical cancer cell lines C-33A and SiHa cell lines. (b) Western blot detected the protein expression level of KNTC1 in human normal cervical epithelial cells (HcerEpic) and cervical cancer cell lines C-33A and SiHa cell lines. KNTC1, kinetochore-associated protein 1.

To validate the results of bioinformatics analysis, we first determined KNTC1 expression in normal human cervical epithelial cells, HcerEpic, and in two cervical cancer cell lines, SiHa and C-33A. Western blotting and PCR were used to detect KNTC1 expression levels in SiHa and C-33A cell lines, and both mRNA and protein expression levels of KNTC1 were upregulated compared to that in the normal human cervical epithelial cell line HcerEpic. Especially in SiHa cells, KNTC1 expression was approximately three times higher than in HcerEpic cells (Figure 2a, b). This showed that the results of bioinformatics analysis were reliable.

### Downregulation of KNTC1 inhibited cell proliferation

**Figure 3.**
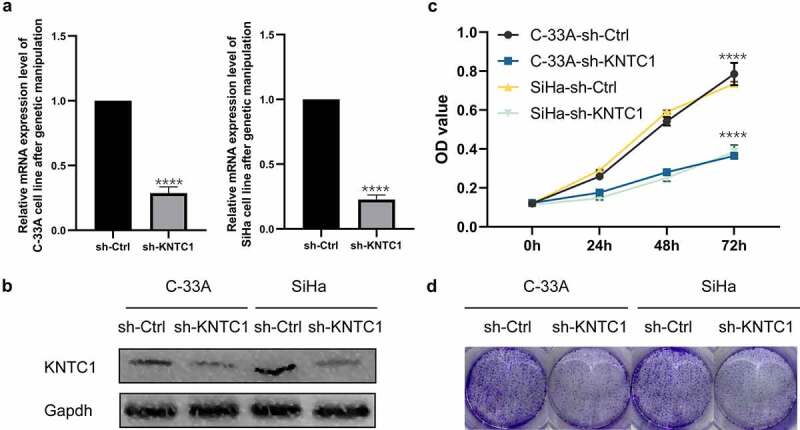
Knockdown of KNTC1 inhibited the proliferation of cervical cancer cells. (a) After knocking down KNTC1, KNTC1 mRNA levels decreased in C-33A and SiHa cells. (b) After knocking down KNTC1, the protein level of KNTC1 decreased in C-33A and SiHa cells. (c) CCK8 detected the effect of KNTC1 knockdown on the proliferation of cervical cancer cells. (d) Colony formation assay was used to detect the effect of KNTC1 knockdown on proliferation of cervical cancer cells. KNTC1, kinetochore-associated protein 1.

After confirming the increased expression of KNTC1 in cervical cancer cell lines, we needed to clarify the role of KNTC1 in cervical cancer. We transfected SiHa and C-33A cells with lentivirus to generate stable cervical cancer cell lines with low KNTC1 expression. Transfection efficiency was verified by qRT-PCR and western blotting. As shown in Figure 3a and 3b, after lentivirus transfection, KNTC1 mRNA expression levels in SiHa cells and C-33A cells decreased by 67% and 75%, respectively, and protein expression levels decreased to a certain extent. We used the CCK8 assay to analyze the effect of low expression of KNTC1 on cell proliferation and found that after KNTC1 knock-down, the number of C-33A and SiHa cells decreased and proliferation rate slowed down. After 72 h, the number of cells in the transfection group was approximately half of that in the control group (Figure 3c). Further, colony formation experiments confirmed that the number of colonies formed by SiHa and C-33A cells with low KNTC1 expression decreased, and their independent survival ability also decreased (Figure 3d). This indicated that KNTC1 inhibition weakened cell proliferation.

### Inhibition of KNTC1 attenuated migration and invasion of cervical cancer cells

**Figure 4.**
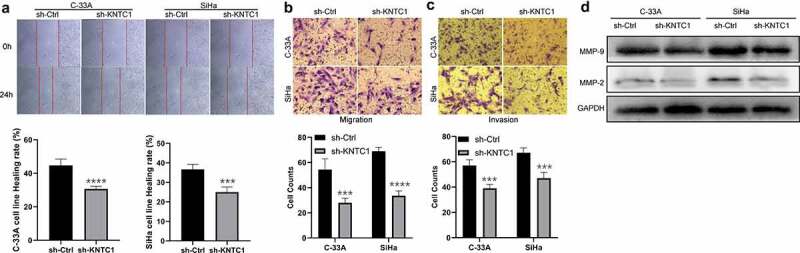
Knockdown of KNTC1 inhibited the migration and invasion of cervical cancer cells. (a) Wound healing experiment was used to detect the effect of KNTC1 knockdown on the migration ability of cervical cancer cells. (b) Transwell assay was used to detect the effect of KNTC1 knockdown on migration of cervical cancer cells. (c) Transwell assay was used to detect the effect of KNTC1 knockdown on invasion of cervical cancer cells. (d) Western blot was used to detect the protein expression levels of MMP2 and MMP9 in cervical cancer cells after knocking down KNTC1. KNTC1, kinetochore-associated protein 1; MMP2, matrix metalloproteinase-2; MMP9, matrix metalloproteinase-9.

We used wound healing and Transwell assays to analyze the role of KNTC1 in migration and invasion of cervical cancer cells. Wound healing experiments showed that cervical cancer cells with low KNTC1 expression had a slower wound healing rate than the shCtrl group (Figure 4a). Transwell experiments showed that KNTC1 knockout significantly reduced the number of migrating SiHa and C-33A cells to only half of that in the control group; the same trend was also observed in the invasion experiment, with the number of cells in the low-expression KNTC1 group being approximately two-thirds of that in the control group (Figure 4b, c). This suggested that KNTC1 knockdown reduces the migration and invasion abilities of cervical cancer cells. The migration and invasion abilities of cells have been reported to be related to the extracellular matrix, and one of the important roles of MMPs is to degrade and reshape the extracellular matrix [22]. MMP2 and MMP9 are important members of the MMP family, which are closely related to tumors and are highly expressed in various tumors. Therefore, western blotting was used to detect the protein expression levels of matrix metallopeptidase 2 (MMP2) and matrix metallopeptidase 9 (MMP9), and after KNTC1 knockdown, both MMP2 and MMP9 were found to be reduced to different extents (Figure 4d). Therefore, we speculated that KNTC1 might regulate the migration and invasion abilities of SiHa and C-33A cells via MMP2 and MMP9.

### Knockdown of KNTC1 inhibited cervical cancer cell development in vivo

**Figure 5.**
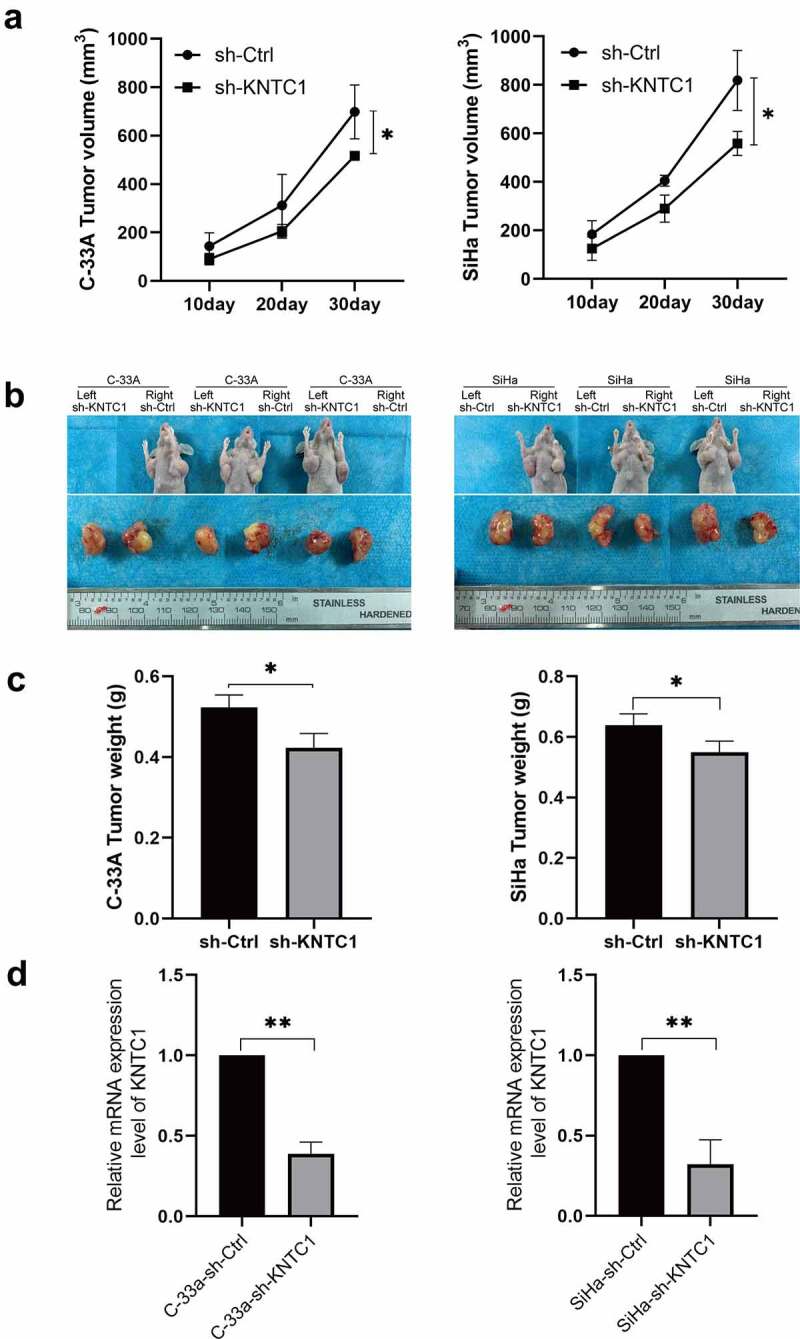
Knockdown of KNTC1 inhibited the tumorigenic ability of cervical cancer cells in vivo. (a) Tumor growth curve in nude mouse model. (b) Tumor display at the end of the experiment (day 30). (c) Comparison of tumor weights at the end of the experiment (day 30). KNTC1, kinetochore-associated protein 1. (d) qPCR results of subcutaneous tumor samples.

KNTC1 promotes the proliferation of cervical cancer cells in vitro. To clarify whether KNTC1 has the same effect in vivo, we used SiHa and C-33A cells to construct a subcutaneous xenograft tumor model in nude mouse. As shown in Figure 5a, after KNTC1 was knocked down in SiHa and C-33A cells, the tumor growth rate slowed down and the volume decreased significantly compared to that in the control group. Subsequently, the mice were sacrificed, and the tumor was removed to verify the efficiency of shRNA operation and observe tumor volume and weight. We found tumor volume and weight in the low-KNTC1 expression group to decrease to a certain extent compared to that in the control group (Figure 5b, c). Subsequently, RNA was extracted for PCR to verify the manipulation efficiency. Results indicated the manipulation to have been successful in both groups, and the knockdown efficiencies of C-33A and SiHa to be 61 and 68%, respectively (Figure 5d). This suggested that the inhibition of KNTC1 retarded tumor proliferation in vivo.

## Discussion

Cervical cancer is a common gynecological tumor, and its prevention and treatment have always been the focus of research. Many molecular markers are currently used to assist the differential diagnosis of cervical neoplasia in the clinic; for example, carcinoma embryonic antigen (CEA), squamous cell carcinoma antigen (SCC-Ag), and CA-125; however, their sensitivity and specificity are not ideal [32]. Patients with early-stage cervical neoplasia can be cured surgically, the trauma of the treatment is relatively severe. However, some patients may have sequelae, such as lymphedema, increased lymphocytes, and urinary incontinence, which affect their quality of life [33,34]. For patients with stage IIB–IVA, the 5-year survival rate of concurrent chemoradiation (CTRT) is 30–80% [35], which increases the incidence of acute toxicities [36]. Therefore, identification of novel targets for early screening and therapy of cervical neoplasia is urgently required.

KNTC1 was first discovered by Roger E. Karess and David M. Glover. The gene was identified to be necessary during normal mitosis in Drosophila. Its mutation affects the process of chromosome transmission to daughter cells during mitosis, thereby causing the cell to exit mitosis early and leading to aneuploidy [37]. In HeLa cells, the KNTC1 protein was found to interact with Zeste-White 10 (ZW10). KNTC1 and ZW10 co-localize in the kinetochore and regulate mitosis [38,39]. Mitosis is closely associated with tumor formation. A normal and ordered mitosis is necessary to maintain genome stability; KNTC1 prevents premature mitosis in cells. If the exit from mitosis is disordered, it will lead to abnormal conditions, such as aneuploidy and aberrant proliferation [40,41], which is one of the common characteristics of tumor cells [42,43].

In our study, we first obtained the differential genes between the normal group and tumor group through bioinformatics, and subsequently, analyzed the differential genes by GO/KEGG enrichment (Figure 1c, d). The results suggested that differential genes mainly play a role in mitosis, cell cycle, DNA replication and separation, and extracellular matrix reconstruction. After combining the heat map results, we chose KNTC1 as the follow-up research focus, since its main function involved cell cycle and DNA replication, and it has not been studied in cervical cancer. We conducted a preliminary study on the prognostic effect of KNTC1 using the TCGA database. KNTC1 showed good sensitivity and specificity and could be better used as a prognostic factor for patients with cervical cancer (Figure 1g); however, results also suggested that there was no statistically significant difference in survival time distribution between the groups (Figure 1h). Subsequently, we explored the role of KNTC1 in the occurrence and development of cervical neoplasia. We found that, compared to that in HcerEpic, a type of normal cervical epithelial cell, KNTC1 was overexpressed in cervical neoplasia cell lines C-33A and SiHa, suggesting that KNTC1 may be upregulated in cervical cancer (Figure 2), which was consistent with previous bioinformatics results (figure 1f). Similar to our findings, KNTC1 had been reported to be upregulated in small cell lung cancer [44]. Prathapan Thiru had found that KNTC1 is upregulated in colorectal cancer, liver cancer, bladder cancer, breast cancer, and other tumors, and is coordinately expressed with other kinetochore proteins, as suggested by comparative datasets [45]. We then knocked down KNTC1 through genetic manipulation and found that, compared to the control group, proliferation and colony-forming abilities were significantly reduced in the knock-down group, hence suggesting that KNTC1 promoted the proliferation of cervical neoplasia. Similar to our study, KNTC1 knockdown in esophageal squamous cell carcinoma [31], colon cancer [46], and bladder cancer [47] had resulted in decreased cell viability and slower proliferation. In addition, in HeLa cells, when KNTC1 was dysfunctional, there were lagging chromosomes and erroneous separation of chromatids during mitosis [38]. Williams et al. isolated ZWILCH from the KNTC1-ZW10 complex in HeLa cells by immunoprecipitation, and discovered the Rod–Zw10–Zwilch complex (RZZ complex) [48]. In HeLa cells, the RZZ complex can mediate the recruitment of Mad1/Mad2 through the N-terminus of KNL1, regulate the cell cycle, and further affect cell proliferation [49].

In our study, the results of wound healing and Transwell assays suggested that, compared to the control group, migration and invasion capabilities declined in the KNTC1 knockdown group, as did protein expression levels of MMP9 and MMP2. KNTC1 has been speculated to facilitate tumor cell migration and invasion by regulating MMP9 and MMP2. This was consistent with the GO/KEGG enrichment results, and the differentially expressed genes were mainly related to the extracellular matrix reconstruction. Upregulation of MMPs leads to extracellular matrix degradation and reconstruction, which is essential for tumor cell migration and invasion. In bladder cancer, KNTC1 knockdown has been reported to reduce tumor cell migration [47]. In addition, bioinformatics results indicated that KNTC1 is a vital gene for the occurrence and development of hepatocellular carcinoma [50], nasopharyngeal carcinoma [51], pancreatic cancer [52], and neuroblastoma [53], and is related to extracellular matrix reconstruction. We further verified the oncogenic effects of KNTC1 using animal experiments. Similar to our results, Huang et al. had reported that in bladder cancer, in-vivo tumor growth was slowed down after knocking down KNTC1 [47]. However, the results of Marina Gonçalves Diniz’s study were contrary to ours. They found that KNTC1 expression was related to tumor size in oral squamous cell carcinoma; compared to small tumors, larger tumors had a lower expression level [54]. Overall, the studies once again suggested that KNTC1 may be associated with tumorigenesis and progression of neoplasms, although by playing different roles. In this study, we demonstrated that KNTC1 is involved in tumorigenesis and invasion. However, the study has a limitation, since it lacked clinical sample validation. In addition, downstream molecular mechanisms would need to be explored in future.

## Conclusion

In this study, we verified the important role of KNTC1 in cervical cancer using bioinformatics analysis. Subsequently, we verified the promotional effect of KNTC1 on cervical neoplasm through in-vivo and in-vitro experiments, and speculated that it could mediate tumor invasion via MMP9 and MMP2. KNTC1 is a highly potent candidate for becoming a therapeutic target for cervical neoplasms in future, and has vital implications in cervical cancer therapy.
